# Morphology Predicts Grade, Transcriptomics Predicts Nodal Status: Task-Dependent Modality Contributions in Multimodal Prostate Cancer Classification

**DOI:** 10.3390/jimaging12090446

**Published:** 2026-09-15

**Authors:** Chae Eun Moon, Ho Jung Song, Yong Suk Kim

**Affiliations:** 1 Department of AI Software Convergence, Konyang University, 158 Gwanjeo-dong-ro, Seo-gu, Daejeon 35365, Republic of Korea; 26810502@konyang.ac.kr; 2 Department of Medical Engineering, Konyang University, 158 Gwanjeo-dong-ro, Seo-gu, Daejeon 35365, Republic of Korea; songhj6692@gmail.com; 3 Department of Artificial Intelligence, Konyang University, 158 Gwanjeo-dong-ro, Seo-gu, Daejeon 35365, Republic of Korea

**Keywords:** prostate cancer, Gleason grading, nodal status, whole-slide imaging, transcriptomics, multimodal deep learning, multiple instance learning, conformal prediction, task-dependent modality contribution

## Abstract

Multimodal studies of prostate cancer typically report aggregate fusion gains from histology and transcriptomics but rarely characterize when each modality is informative. We asked whether morphology and transcriptomics contribute differently to distinct clinical endpoints, using Gleason grading and nodal status prediction. On 401 TCGA-PRAD patients with matched whole-slide images, bulk RNA sequencing, and clinical data, we evaluated 45 morphological configurations, seven RNA configurations, and six fusion strategies across four grading formulations, T-stage, and N-stage. To avoid per-task model selection, morphology used a single fixed configuration (gated ABMIL on UNI features). Contribution estimates used repeated stratified five-fold cross-validation, with a locked conformal-prediction protocol and gene set enrichment analysis. The two endpoints showed opposite modality dependence. For Gleason grade, the fixed morphological model exceeded the best RNA configuration across all 45 configurations (five-class macro-F1: 0.445 versus 0.398). For nodal status, no morphological configuration exceeded AUROC 0.62, whereas transcriptomics reached 0.68 (permutation *p* = 0.037); a direct interaction test confirmed the reversal (bootstrap 95% CI excluding zero). Fusion gains were modest and task-dependent; enrichment analysis linked the nodal signal to loss of smooth muscle programs, consistent with established dedifferentiation biology. The informative modality is task-dependent: morphology predicts grade, transcriptomics predicts nodal status.

## 1. Introduction

Prostate cancer is one of the most frequently diagnosed malignancies and a leading cause of cancer-related death in men worldwide [1]. Clinical management depends heavily on accurate risk stratification, for which the Gleason grading system remains the cornerstone. This system assigns a grade group (GG 1 to 5) based on the architectural pattern of tumor glands, and it directly informs decisions ranging from active surveillance to radical treatment [2,3]. Despite its central role, Gleason grading is inherently subjective and suffers from substantial inter-observer variability, with agreement among expert uropathologists, measured by quadratic weighted kappa (QWK), typically around 0.79 [4,5]. This ceiling on human reproducibility has motivated objective computational grading tools, and it also sets a realistic reference point against which automated systems should be judged.

The digitization of histopathology into whole-slide images (WSIs) has enabled deep learning-based automated Gleason grading [6]. Because a WSI is a gigapixel image with only slide-level labels, the task is typically formulated as multiple instance learning (MIL), in which a frozen feature extractor embeds each tissue patch and an aggregation module combines these embeddings into a slide-level representation [7]. Pathology foundation models such as UNI, Virchow2, Phikon, and H-optimus-0 now provide strong patch embeddings [8,9,10,11,12], and attention-based aggregators, including ABMIL, CLAM, DSMIL, and TransMIL, weight diagnostically informative regions [7,13,14,15]. On this basis, state-of-the-art systems now approach or match expert pathologists on grading, reporting QWKs up to 0.93 internally and 0.80 to 0.96 externally, while benchmarks show that a strong feature extractor leaves comparatively little variance to the aggregator [16,17,18]. The raw grading performance problem is thus largely solved on the tasks and cohorts studied, which shifts scientific interest from headline accuracy toward understanding what each modality and design choice actually contributes, and under what conditions.

Morphology alone, however, does not capture the full biology of a tumor. Bulk RNA sequencing reflects molecular programs such as proliferation, differentiation, and stromal composition, many of which have no unambiguous morphological correlate [19]. This complementarity has motivated extensive multimodal work that integrates WSIs with transcriptomic data for prognosis and risk stratification, using fusion strategies that range from simple late fusion to co-attention and tensor-based interaction models such as Pathomic Fusion and MCAT [20,21,22,23,24,25]. Despite this methodological richness, two gaps persist. First, most studies foreground the performance gain from fusion but provide limited analysis of when and why fusion helps, and several careful analyses report that naive fusion does not consistently exceed the strongest single modality, especially at modest sample sizes [23,26]. Second, the relative contribution of morphology versus molecular data is often treated as fixed, whereas it may depend strongly on the endpoint being predicted, tested within a single cohort under a broad, systematically evaluated set of configurations. It is precisely this task-dependent structure, and its biological rationale, that the present study sets out to characterize.

Prostate cancer is a particularly well-suited setting for this question, because two clinically central endpoints are grounded in fundamentally different biology. Tumor differentiation, captured by the Gleason grade, is by construction a morphological quantity: the grading system is defined by glandular architecture, with low grades preserving discrete, well-formed glands and higher grades identified by the progressive loss of the ability to form separate glands [2,3]. Grade is thus read almost by definition from tissue morphology, so image-based models are expected to predict it well. In contrast, the propensity for lymph node metastasis (nodal status, or N-stage) reflects invasive and disseminative capacity, governed by molecular and microenvironmental programs that need not leave a discrete, gland-level architectural signature in the primary tumor. These two endpoints therefore offer a natural test of whether morphology and transcriptomics dominate different prediction tasks.

Both sides of this contrast are supported by prior evidence. On the morphological side, direct attempts to predict nodal metastasis from primary prostate histology have achieved only modest performance: Wessels et al. trained a convolutional neural network on 218 patients matched for established risk factors, including Gleason score, tumor size, venous and perineural invasion, and age, so that the model had to find signals beyond established grade features, yet reached a mean AUROC of only 0.68, with 53% sensitivity and 70% specificity [27]. That a dedicated model, even after controlling for grade, recovered only weak signals, and in particular missed roughly half of node-positive cases, underscores that nodal status is not primarily a morphological readout. On the molecular side, prostate cancer progression is accompanied by a well-documented remodeling of the tumor stroma, in which the normal smooth muscle compartment is progressively lost and replaced by a reactive, myofibroblast-rich stroma; this loss of smooth muscle and contractile programs has been characterized histologically and transcriptomically over two decades and linked to higher grade, metastasis, and adverse outcomes, including in recent large-scale and single-cell analyses [28,29,30]. Because this shift is a change in gene-expression state that is only partially reflected in glandular morphology, it is biologically plausible that metastatic propensity is legible to transcriptomics while remaining largely illegible to image-based grading.

Two methodological choices shape how such a contrast should be established. First, most grading and fusion studies lack rigorous uncertainty quantification, yet a point prediction is most dangerous exactly where models are least reliable, on minority or borderline cases; conformal prediction addresses this by returning, for each case, a prediction set guaranteed to contain the true label at a user-specified coverage, allowing the model to abstain on ambiguous inputs [31,32]. Second, characterizing modality contributions is demanding on the evaluation itself, since with a few hundred patients a single held-out split can distort the apparent ranking of modalities, especially for ordinal metrics such as QWK. A locked protocol yields an unbiased final estimate but with high variance, whereas repeated stratified five-fold cross-validation uses every patient for more stable estimates; we therefore report both and treat their discrepancy as itself informative about the reliability of small-cohort evaluation.

In this study, we present a rigorously evaluated multimodal analysis of prostate cancer on the TCGA-PRAD cohort, comprising 401 patients with matched WSIs, RNA-seq, and clinical annotations, designed to characterize task-dependent modality contributions rather than to maximize any single performance metric. Our contributions are fourfold:

(1) Task-dependent modality contributions. Under a consistent cross-validation evaluation, Gleason Grade Group is predicted substantially better by morphology (attention-based MIL) than by transcriptomics, whereas nodal status is predicted by transcriptomics but not reliably by morphology, with no configuration exceeding an AUROC of 0.62, consistent with prior reports of limited morphological predictability of nodal status and with the known molecular basis of prostate cancer progression.
(2) Systematic robustness. For each task, we evaluate 45 morphological configurations (9 aggregators × 5 foundation models) and seven RNA preprocessing and model combinations, showing that this contrast is not an artifact of a particular feature extractor, aggregator, or preprocessing choice.
(3) Dual evaluation protocol. We report both a held-out locked protocol and repeated stratified five-fold cross-validation and analyze their discrepancy, showing that a small single test set can substantially misestimate modality contributions and, in particular, understate the strength of morphology for grading.
(4) Uncertainty-aware prediction. We integrate conformal prediction to provide calibrated prediction sets, quantifying per-case reliability for clinically actionable risk groups.

## 2. Materials and Methods

**Figure 1:**
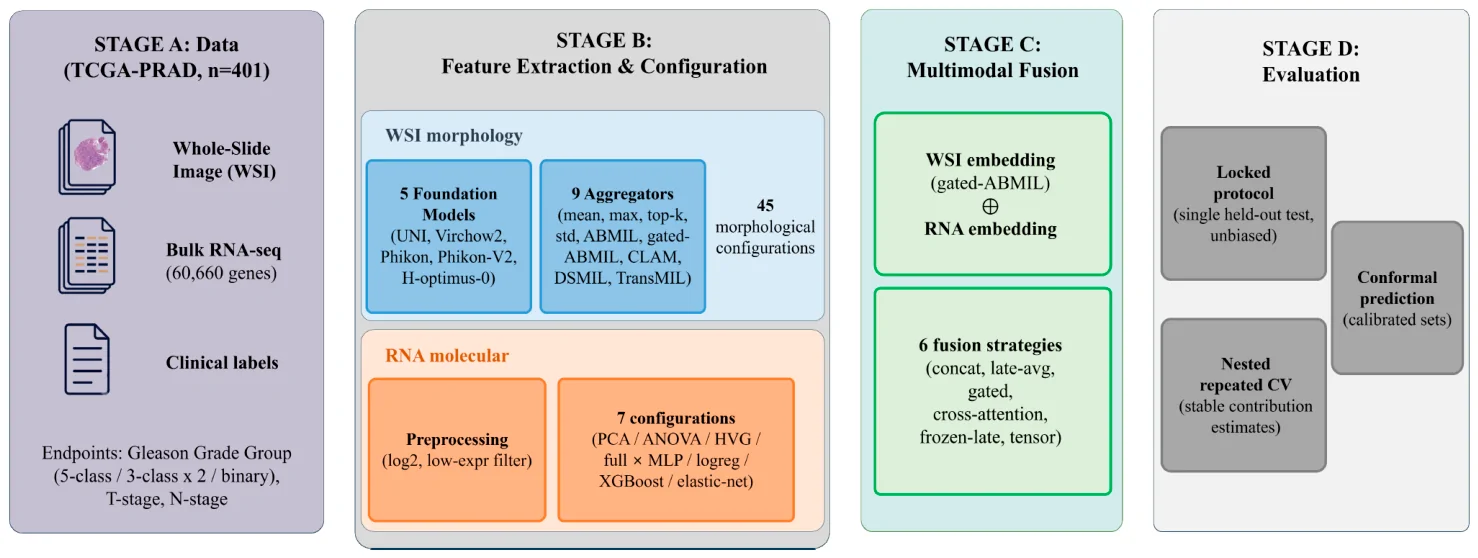
Overview of the multimodal analysis pipeline for prostate cancer on the TCGA-PRAD cohort (*n* = 401). (**STAGE A**) Data construction: patients with a matched whole-slide image (WSI), bulk RNA-seq (60,660 genes), and clinical annotations were assembled, defining six classification endpoints (Gleason Grade Group in five-class, two three-class, and binary formulations; pathologic T-stage; and pathologic N-stage). (**STAGE B**) Feature extraction and configuration, performed independently for each modality: the morphological branch combines five pathology foundation models with nine aggregation strategies, yielding 45 configurations per task, while the molecular branch combines RNA feature representations and classifier families into seven configurations. (**STAGE C**) Multimodal fusion of the best unimodal WSI encoder with the RNA embedding under six fusion strategies of increasing expressiveness, benchmarked against matched unimodal baselines. (**STAGE D**) Dual evaluation: a held-out locked protocol providing an unbiased single estimate and repeated stratified five-fold cross-validation providing stable contribution estimates, complemented by conformal prediction for calibrated prediction sets. All feature selection and dimensionality reduction was performed within training folds only.

The overall pipeline, including cohort construction, per-modality feature extraction and configuration, multimodal fusion, and the dual evaluation protocol, is illustrated in Figure 1. Each component is detailed in the following subsections.

### 2.1. Dataset and Endpoints

This study used the publicly available Cancer Genome Atlas Prostate Adenocarcinoma (TCGA-PRAD) cohort [33]. We assembled 401 patients for whom a diagnostic whole-slide image (WSI), matched bulk RNA sequencing, and clinical annotations were all available. All three data types (WSI, RNA-seq, and clinical annotation) were matched at the patient level by TCGA case identifier, and only patients for whom all modalities were available were included, so that every multimodal sample contains correctly paired measurements from the same patient and tumor specimen. For patients with more than one WSI, a single slide was retained per patient. Bulk RNA-seq was obtained as transcripts per million (TPM) values for 60,660 genes.

We considered six classification endpoints derived from the same cohort, summarized in Table 1: four Gleason grading formulations, pathologic T-stage, and pathologic N-stage. The primary morphological endpoint was the ISUP Grade Group (GG), evaluated in three formulations to probe the ordinal structure of grades: a five-class task (GG 1 to 5); a three-class task grouping GG as {1}, {2,3}, {4,5} (denoted 1/23/45); and an alternative three-class task grouping GG as {1,2}, {3}, {4,5} (denoted 12/3/45), which isolates the diagnostically ambiguous GG 3 as its own class. A binary high-grade task (GG {1,2,3} vs. {4,5}) was also evaluated. The molecular endpoints were pathologic T-stage (T2 vs. T3 and above; *n* = 373 with available labels) and pathologic N-stage, that is, regional lymph node status (N0 vs. N1; *n* = 316 with available labels, of which 47 were N1). N-stage labels were available only for patients who underwent lymph node dissection, so this subset of 316 constitutes the maximum evaluable cohort for that endpoint, a constraint we return to when interpreting statistical power. Beyond Gleason grade and pathologic stage, additional clinical covariates such as preoperative prostate-specific antigen (PSA), age, and surgical margin status were not used as model inputs or adjustment variables, consistent with the study’s focus on the imaging-versus-transcriptomic contrast for the two anchor endpoints.

Among these endpoints, Gleason grade and nodal status serve as the two contrasting anchors for testing task-dependent modality contribution, because they differ in biological nature: Gleason grade is defined directly by glandular architecture and is therefore expected to be morphologically legible, whereas nodal status reflects invasive and disseminative capacity that is governed by molecular programs and need not have a clear morphological correlate. T-stage, an intermediate measure of local tumor extent, was included as a reference endpoint expected to lie between these extremes.

### 2.2. Feature Extraction, Modality Configurations and Fusion

A central design principle of this study is to characterize each modality as thoroughly and symmetrically as possible, rather than committing to a single favored configuration, so that any observed modality contrast can be attributed to the intrinsic information content of morphology or transcriptomics rather than to an incidental modeling choice. We therefore evaluated a broad grid of configurations for both modalities, summarized in Table 2, applying each configuration to all six endpoints (the four Gleason grading formulations, T-stage, and N-stage). All feature selection and dimensionality reduction was performed strictly within training folds to prevent leakage.

For the morphological modality, tissue regions in each WSI were segmented on a 32× downsampled thumbnail by thresholding the saturation channel of the HSV color space, and the slide was tiled into non-overlapping 256 × 256-pixel patches at the highest magnification, retaining patches with at least 20% tissue. Each patch was resized to 224 × 224 and normalized with ImageNet statistics, and patch-level features were extracted using five frozen pathology foundation models spanning a range of architectures and pretraining corpora: UNI (1024-dimensional), Virchow2 (2560-dimensional), Phikon (768-dimensional), Phikon-v2 (1024-dimensional), and H-optimus-0 (1536-dimensional) [8,9,10,11,12]. No fine-tuning was applied, so that the comparison isolated the quality of the pretrained representation, and up to 2000 patches were sampled per slide for the learnable aggregators to bound computation. We then evaluated nine aggregation strategies for each of the five feature extractors, yielding 45 morphological configurations per task. The aggregators comprised four non-parametric pooling operators (mean, max, top-k mean, and standard deviation), three attention-based multiple instance learning (MIL) models (ABMIL, gated ABMIL, and CLAM), one dual-stream model (DSMIL), and one Transformer-based model (TransMIL) [7,13,14,15]. This spectrum, ranging from parameter-free pooling through learned attention to self-attention, ensures that if morphology yields only a weak predictive signal for a given endpoint across all 45 configurations, this reflects a genuine limitation of tissue architecture for that endpoint rather than the shortcoming of any single aggregator. Each aggregation strategy maps the variable-length set of patch features from a slide to a single fixed-length patient-level representation, so that all downstream modeling and evaluation operates at the patient level.

For the molecular modality, RNA-seq TPM values were log2-transformed after adding a pseudocount, and low-expression genes were removed using a training-fold median threshold. Gene-level standardization and all feature selection were computed on training-fold data only and applied to the held-out fold, so that no test-fold statistics entered preprocessing. To characterize transcriptomics as thoroughly as morphology, and to preempt the concern that a single preprocessing or model choice might understate RNA performance, we evaluated seven RNA configurations spanning four feature representations, namely, principal component analysis (PCA, 128 components), analysis-of-variance (ANOVA) F-test selection of the top 2000 genes, selection of the top 2000 high-variance genes, and the full standardized gene set, paired with classifier families, including a multilayer perceptron (MLP), L2-regularized logistic regression, gradient-boosted trees (XGBoost), and an elastic-net linear model (Table 2). This grid spans linear and non-linear models as well as unsupervised and supervised feature selection, so that the resulting RNA performance represents a fair upper estimate rather than an artifact of one pipeline.

Finally, to test whether combining the two modalities exceeds the stronger single modality, and which fusion mechanism is most effective at this cohort size, we compared six fusion strategies of increasing expressiveness. Because fusion multiplies the number of trainable configurations, we restricted the fusion experiments to the components that the unimodal analysis had identified as most informative, rather than repeating the full 45-configuration grid. Specifically, fusion was performed on UNI features, which gave the strongest and most consistent morphological performance across tasks, and on the two attention-based slide encoders that led the unimodal comparison, ABMIL and gated ABMIL. Five strategies, namely, concatenation, late fusion by probability averaging, gated fusion, feature-level cross-attention, and a frozen late-fusion scheme, were evaluated with both encoders, and a low-rank tensor (Kronecker-product) fusion was additionally evaluated with the best encoder [22,24,25]. In every case, the two modalities were encoded independently before fusion: the WSI branch produced a slide-level embedding through the attention MIL encoder on UNI features, and the RNA branch produced a matched embedding through its own encoder, each projected to a common 256-dimensional representation. The strategies differ in where the two representations are combined: probability averaging and frozen late fusion combine them at the decision level, whereas concatenation, gated fusion, cross-attention, and low-rank tensor fusion combine them at the feature level before a shared classification head. All embeddings were computed within training folds. For a fair comparison, unimodal WSI-only and RNA-only predictions were obtained from the same embeddings and folds, so that any fusion gain is measured against a matched baseline rather than a separately optimized one.

Model hyperparameters. All aggregators and classifiers used compact architectures to limit overfitting on this cohort size. The pooling-based and RNA multilayer-perceptron classifiers used a single hidden layer of 128 units with ReLU activation and a dropout of 0.3, trained with the Adam optimizer (learning rate: 1 × 10^−3^, weight decay: 1 × 10^−3^) for up to 200 epochs with a class-balanced focal loss. The attention-based slide encoders (ABMIL, gated ABMIL, CLAM, DSMIL, and TransMIL) used a hidden dimension of 128 with a dropout of 0.3, trained with Adam (learning rate: 1 × 10^−4^, weight decay: 1 × 10^−3^) for 25 to 30 epochs on up to 2000 patches per slide sampled from UNI features. For the transcriptomic configurations, feature reduction used either principal component analysis (128 components), supervised ANOVA-based selection of the top 2000 genes, or high-variance selection of the top 2000 genes; classifiers were the same 128-unit multilayer perceptron, a logistic-regression model (Adam; learning rate: 1 × 10^−2^, weight decay: 1 × 10^−2^), an elastic-net linear model (L1: 1 × 10^−4^, L2: 1 × 10^−3^), and gradient boosting (XGBoost; maximum depth: 4, learning rate: 0.1, subsample: 0.8, column subsample: 0.5, minimum child weight: 3, 100 boosting rounds). For fusion, each modality was first encoded to a 256-dimensional representation (RNA branch trained with AdamW, learning rate: 1 × 10^−3^), and the fusion head was trained with AdamW (learning rate: 5 × 10^−5^, weight decay: 1 × 10^−5^) for 100 epochs; the low-rank tensor-fusion variant used rank 16. Class imbalance was addressed throughout by class-balanced loss weighting.

### 2.3. Evaluation

Characterizing modality contributions places high demands on the evaluation itself, because with a cohort of a few hundred patients a single data split can distort the apparent ranking of modalities. We therefore used two complementary protocols with distinct roles. All analyses aimed at characterizing modality contributions: the 45 morphological configurations, the seven RNA configurations, the six fusion strategies, and the unimodal T-stage and N-stage models were evaluated by repeated stratified five-fold cross-validation, in which every patient contributes to evaluation across repeated fold assignments and all model and feature selection is performed strictly within the training folds. Because patch-level features are aggregated into a single patient-level representation before any partitioning, every patch from a given patient is inseparable and necessarily falls in the same fold; a patch-level split, which could inflate performance by placing highly similar tissue regions from one patient in both training and test sets, is therefore structurally impossible in our design. All splits, model fitting, feature selection, standardization, and dimensionality reduction operate at the patient level within training folds only. This protocol yields stable contribution estimates that are not driven by a single fortunate or unfortunate split. Separately, an unbiased final estimate of grading performance, together with conformal calibration, was obtained from a held-out locked protocol, in which the cohort was partitioned once into training, validation, calibration, and test sets (280, 40, 40, and 41 patients for the grading tasks) and the test set was evaluated only a single time. Reporting both protocols allows their discrepancy to be examined, since a large gap between a single locked estimate and a stable cross-validated estimate is itself evidence about the reliability of small-cohort evaluation. To prevent information leakage, all feature selection, dimensionality reduction, standardization, and low-expression filtering (retaining genes with a training-fold median log2 expression above 0.1) was performed inside the training portion of each fold.

The cross-validation protocol was as follows. For each endpoint, patients were partitioned into five stratified folds, with stratification on the five-class Gleason label so that every grade group was represented in each fold; the same fold structure was reused across endpoints. Each configuration was trained on four folds and evaluated on the held-out fold, cycling through all five folds so that every patient received exactly one out-of-fold prediction. This procedure was repeated five times with different random seeds (base seed: 2024) for the pooling, RNA, and cascade classifiers, and twice for the more computationally intensive attention-based and fusion models. Out-of-fold predicted probabilities were averaged across repeats at the patient level before computing metrics. Because hyperparameters were fixed a priori (Section 2.2) rather than tuned, the classifier evaluation is a repeated stratified five-fold cross-validation and does not require an inner tuning loop.

For the transcriptomic nodal-status signature, feature selection was performed with a fully nested procedure to prevent leakage: within the training partition of each outer fold, an inner five-fold, five-repeat cross-validation selected the most reproducible differentially expressed genes (top 200 per inner fold by Welch t statistic, retaining genes selected in at least 80% of inner folds), and only these training-derived genes were used to fit the nodal classifier before evaluation on the untouched outer test fold. All normalization (fold-specific z-scoring) and dimensionality reduction was likewise computed on training data only. No test-fold information entered the gene filtering, signature selection, normalization, or model fitting.

Performance was quantified with metrics matched to each endpoint. For the ordinal Gleason grading tasks, we report accuracy, macro-averaged F1-score, quadratic weighted kappa (QWK), mean absolute error (MAE) on the ordinal scale, and the number of severe errors, defined as predictions differing from the true grade by two or more grade groups. Macro-F1 was treated as the primary classification metric and QWK as the primary ordinal metric, reflecting the class-balanced, order-aware evaluation appropriate to imbalanced grade distributions. Because macro-F1 is the unweighted mean of the per-class F1 scores, it weights minority grade groups equally with majority ones, giving class-specific sensitivity to rare high-grade classes without a separate per-class table. For the binary T-stage and N-stage endpoints, discrimination was summarized by the area under the receiver operating characteristic curve (AUROC) with 95% confidence intervals, and operating points were reported as sensitivity and specificity pairs. We additionally report the Matthews correlation coefficient (MCC), which is robust to class imbalance.

For the staging endpoints, each modality was summarized by a single representative model to enable a like-for-like comparison: WSI by a PCA-reduced logistic model on UNI features, RNA by a logistic model on principal components of the full standardized gene set, and grade by the pathologic grade group. These representative staging estimates are computed on the primary staging protocol and are distinct from the 45-configuration morphological grid, which we report in the Results; the two analyses agree in showing that morphology does not reliably predict nodal status.

Finally, to equip the grading classifier with calibrated uncertainty rather than point predictions alone, we applied split conformal prediction with adaptive prediction sets (APSs) on the locked protocol, whose dedicated calibration partition was used to compute nonconformity scores [31,32]. Prediction sets were formed at user-specified target coverage levels, and we report empirical coverage against the target together with the mean prediction-set size, where, at a fixed coverage, a smaller set indicates a more informative and confident predictor. This provides a per-case, interpretable measure of reliability that is particularly relevant for minority and borderline cases.

### 2.4. Statistical Validation and Biological Interpretation

Because the central claim of this study is a contrast in modality contributions rather than a single performance number, we performed a series of controls to ensure that the observed contrast was not an artifact. The statistical significance of modality differences was assessed by permutation and bootstrap procedures, with confidence intervals reported alongside point estimates. Task-dependence itself was tested directly as an endpoint-by-modality interaction, defined as the difference between the transcriptomics-minus-morphology AUROC gap on the nodal endpoint and the same gap on the T-stage endpoint; a non-zero interaction indicates that the relative contribution of the two modalities reverses across tasks rather than reflecting a uniform modality advantage. Point estimates are reported with bootstrap confidence intervals and permutation *p*-values; the interaction result is reported in Section 3.1. Given the limited number of node-positive cases, boundary results were interpreted conservatively.

Three potential confounders specific to the molecular N-stage signal were addressed directly. First, to exclude confounding by tumor purity, the N-stage analysis was repeated after adjusting for a stromal- and immune-marker-based purity score computed in the ESTIMATE framework, verifying that the transcriptomic N-stage signal (point-biserial correlation between purity and N-stage, r = −0.03) persisted independently of tumor cellularity. Second, to exclude batch effects arising from the multi-institutional nature of TCGA, the association between tissue source site (TSS) and N-stage was tested by a chi-square test. Third, to determine whether the molecular N-stage signal was merely a proxy for grade, N-stage prediction was re-evaluated after controlling for Gleason grade, confirming that the transcriptomic signal was at least partially independent of grade.

To provide a biological interpretation of the transcriptomic N-stage signal, gene set enrichment analysis (GSEA) was performed on the differential-expression ranking between node-positive and node-negative tumors [34]. We emphasize that this analysis serves to connect our observations to established stromal and dedifferentiation biology in prostate cancer and do not claim that these mechanisms are novel; the smooth muscle and contractile programs discussed in the Introduction have been characterized in prior work, and our enrichment analysis is used only to verify that the same programs underlie the molecular signal we detect.

All classifiers and dimensionality-reduction steps were implemented within a single consistent codebase. Principal component analysis was computed by singular value decomposition, and neural components were trained with the Adam or AdamW optimizer using class-balanced weighting to address label imbalance. Foundation-model feature extraction was performed on GPU. The number of training epochs for the learnable aggregators was chosen following an epoch-sensitivity analysis, which identified an early-stopping point appropriate to the cohort size and confirmed that the reported configurations were neither underfit nor overfit.

Reproducibility. All experiments were implemented in Python 3.10 with PyTorch 2.13, scikit-learn 1.7, NumPy 2.2, SciPy 1.15, and XGBoost 3.2; gene set enrichment analysis used gseapy 1.3. Foundation-model feature extraction and all model training were performed on a single NVIDIA L4 GPU (NVIDIA Corporation, Santa Clara, CA, USA). Random seeds were fixed for cross-validation fold assignment and model initialization to make the reported results reproducible. The analysis code and trained model weights are available from the corresponding author upon reasonable request, consistent with the Data Availability Statement. The computational cost is modest and dominated by one-time foundation-model feature extraction from whole-slide images; once patch features are precomputed, all downstream components are lightweight. The trained classifiers are small: the logistic-regression heads on PCA-reduced features have on the order of a few hundred parameters, the multilayer-perceptron classifiers with a single 128-unit hidden layer have on the order of 10^4^ to 10^5^ parameters, and the gated-attention MIL encoder (mapping 1024-dimensional UNI features through a 128-unit attention module and classifier) has on the order of 10^5^ parameters. The transcriptomic and slide-level classifiers and the fusion head train in seconds to a few minutes per cross-validation repeat on the single L4 GPU, and the attention-based multiple-instance encoders, which process up to 2000 patch embeddings per slide, are the most demanding of the trainable components but still complete within minutes per fold. Inference for any configuration is near-instantaneous relative to feature extraction. Adding the transcriptomic branch to a morphological model therefore introduces negligible additional training or inference cost, so the multimodal setup does not impose a substantial practical overhead over a unimodal one.

## 3. Results

### 3.1. Task-Dependent Modality Contributions

Prostate cancer allows a direct test of whether morphology and transcriptomics contribute differently to different endpoints, because Gleason grade is a morphologically defined quantity, whereas nodal status reflects a molecular state. We first examined the four Gleason grading formulations and then the two staging endpoints, evaluating each modality under repeated stratified five-fold cross-validation so that contribution estimates were not driven by a single data split. For the grading tasks, morphology was represented by a single fixed configuration, gated ABMIL on UNI features, chosen in advance to avoid selecting the most favorable morphological model for each task, and compared against the strongest of the seven RNA configurations. Across all four formulations, this fixed morphological model exceeded the best RNA configuration on every metric.

The magnitude of this advantage is shown in Table 3. On the five-class task, the fixed morphological model reached a macro-F1 of 0.445 and a QWK of 0.613 versus 0.398 and 0.591 for the best of the seven RNA configurations. Morphology also led on both three-class formulations (macro-F1: 0.637 versus 0.618, QWK: 0.619 versus 0.536 for 1/23/45; macro-F1: 0.603 versus 0.599, QWK: 0.633 versus 0.606 for 12/3/45). The one exception was the binary high-grade task, the easiest and most collapsed endpoint, where the two modalities were essentially tied (macro-F1: 0.799 versus 0.802). Thus, morphology holds a consistent advantage on the finer-grained ordinal grading tasks, which are the ones that most depend on glandular architecture, while the advantage disappears only when the grading task is reduced to a single high-versus-low threshold.

The Matthews correlation coefficient (MCC), which is robust to class imbalance, shows the same overall pattern: morphology matches or exceeds transcriptomics on the two three-class formulations and the binary task (for example, 0.521 versus 0.497 on the 1/23/45 grouping and 0.622 versus 0.604 for the binary task), while on the five-class task the two modalities are essentially tied (0.285 versus 0.297). This is consistent with the macro-F1 and QWK results and reinforces that the morphological advantage is clearest on the finer-graded distinctions rather than on the hardest five-class formulation.

**Figure 2:**
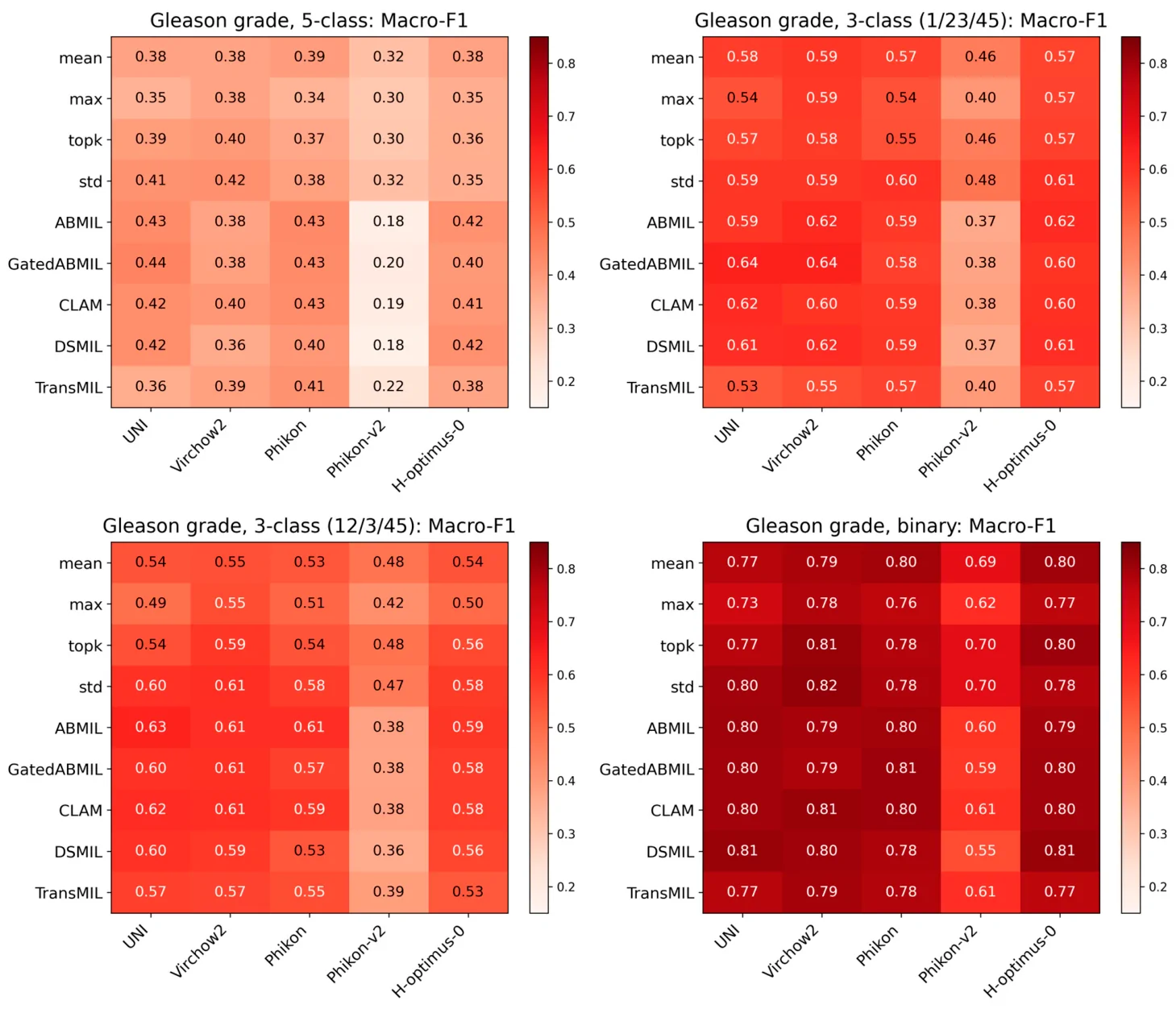
Morphology predicts Gleason grade across all configurations. Each heatmap shows the repeated stratified five-fold cross-validation macro-F1 for the 45 morphological configurations (9 aggregation strategies, rows; 5 foundation models, columns) on one Gleason grading formulation: five-class (**top left**), three-class 1/23/45 (**top right**), three-class 12/3/45 (**bottom left**), and binary high-grade (**bottom right**). Attention-based multiple instance learning (ABMIL, gated ABMIL, and CLAM) generally leads over non-parametric pooling, and performance is high across most configurations, in contrast to the near-chance N-stage performance of the same pipeline (Figure 3). Phikon-v2 is a consistent outlier, performing poorly under learnable aggregators. All values are macro-F1 under repeated stratified five-fold cross-validation on the full cohort (n = 401).

This morphological advantage held across the full configuration grid rather than being confined to the best model (Figure 2). Attention-based multiple instance learning (ABMIL, gated ABMIL, and CLAM) generally led over non-parametric pooling, and macro-F1 was high across most of the 45 configurations for every grading formulation. Phikon-v2 was a consistent outlier, performing markedly worse than the other foundation models under learnable aggregators. These results establish that Gleason grade, a morphologically defined endpoint, is best predicted from morphology, robustly across feature extractors and aggregators.

The staging endpoints reversed this pattern (Table 4). Using AUROC under repeated stratified five-fold cross-validation, morphology did not reliably predict N-stage: the WSI model reached only 0.574 (95% CI: 0.473 to 0.667), a confidence interval that includes chance, whereas transcriptomics reached 0.683 (0.601 to 0.761) and the Gleason grade itself reached 0.687 (0.604 to 0.769). A permutation test confirmed that the RNA advantage over morphology for N-stage was significant (*p* = 0.037). In contrast, T-stage was captured comparably by both modalities, with WSI at 0.770, RNA at 0.716, and Gleason grade at 0.741. Thus, morphology predicts local tumor extent but not nodal status, while transcriptomics is required for the latter.

These conclusions are unchanged under metrics designed for class imbalance. For the imbalanced nodal endpoint (14.9% positive), the area under the precision–recall curve was higher for transcriptomics (0.260) than for morphology (0.199), with the morphological value barely exceeding the random baseline equal to prevalence (0.149); balanced accuracy showed the same ordering (0.67 versus 0.60). Gleason grade used as a predictor performed comparably to transcriptomics on the nodal endpoint (PR-AUC: 0.226, balanced accuracy: 0.74), consistent with grade being a partly morphological summary; transcriptomics nonetheless carried nodal information beyond grade, as shown by the grade-adjusted analysis. For T-stage, the pattern reversed, with morphology achieving the highest PR-AUC (0.844 versus 0.789 for RNA). Thus, the task-dependent contrast holds under precision–recall analysis and balanced accuracy, not only under AUROC.

**Figure 3:**
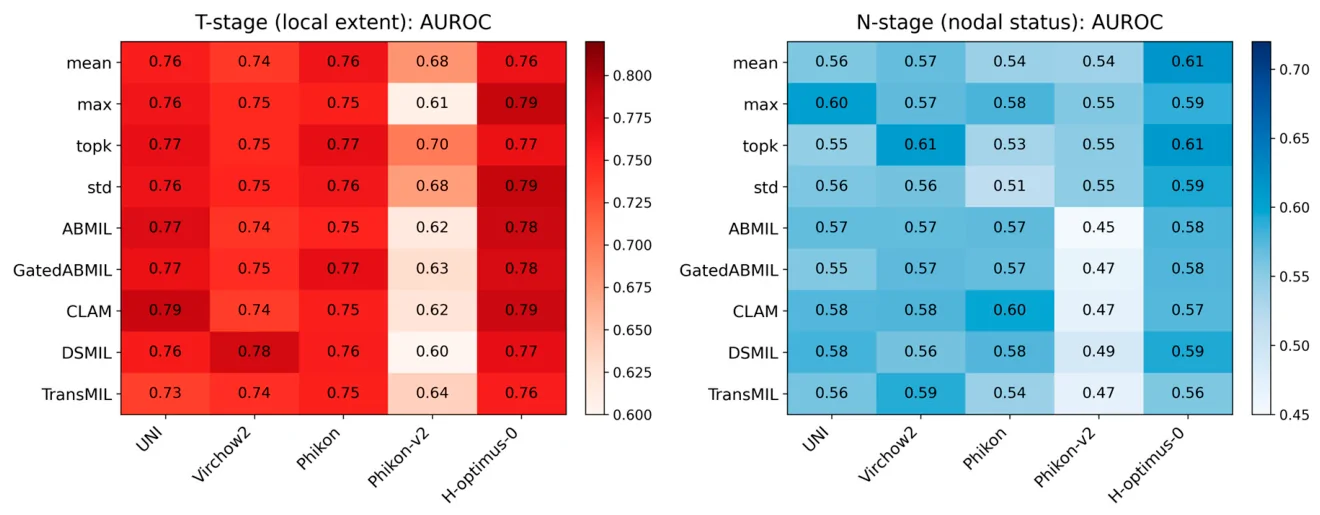
Morphology predicts local tumor extent but not nodal status. Each heatmap shows the repeated stratified five-fold cross-validation AUROC for all 45 morphological configurations (9 aggregation strategies, rows; 5 foundation models, columns). (**Left**) T-stage, where most configurations reach AUROC 0.73 to 0.79. (**Right**) N-stage, where no configuration exceeds 0.62 and most cluster near 0.5 (chance), regardless of foundation model or aggregation strategy. The same imaging pipeline that captures local tumor extent is uninformative for nodal status, indicating an endpoint-specific limitation rather than a weakness of any single model. Phikon-v2 is a weaker feature extractor across both endpoints. Values are AUROC under repeated stratified five-fold cross-validation; T-stage was evaluable in 373 patients (149 T2, 224 T3+) and N-stage in 316 patients (269 N0, 47 N1).

As with grading, this contrast was not an artifact of a particular model (Figure 3). Across all 45 morphological configurations, N-stage AUROC ranged only from 0.45 to 0.61, with no foundation model or aggregation strategy exceeding 0.62, whereas most of the same configurations reached roughly 0.73 to 0.79 for T-stage (with the weaker Phikon-v2 features lower). That the identical imaging pipeline is informative for T-stage but not N-stage indicates an endpoint-specific limitation rather than a general weakness of the imaging models. This endpoint-specific pattern is confirmed by a direct endpoint-by-modality interaction test: transcriptomics outperformed morphology on the nodal endpoint (AUROC gap: +0.106; permutation *p* = 0.037), whereas morphology was at least as strong on T-stage (gap: −0.054), giving a reversal interaction of +0.159 (bootstrap 95% CI: +0.026 to +0.292, excluding zero; +0.132 by a label-permutation test, *p* = 0.052). We regard the direction of the reversal as robust and its statistical strength as moderate given the limited number of node-positive cases. The direction of this contrast was consistent across the repeated fold assignments and random seeds used in cross-validation, since each reported estimate is already averaged over multiple folds and repeats, and it was independently reproduced under the held-out locked protocol. The contrast therefore does not depend on a particular data partition, model configuration, or seed.

### 3.2. Multimodal Fusion

We next asked whether combining morphology and transcriptomics improves prediction over the stronger single modality and which fusion mechanism is most effective at this cohort size. For the grading tasks, we fused the gated ABMIL (UNI) slide embedding with a matched RNA embedding under six fusion strategies, benchmarking each against the WSI-only and RNA-only predictions obtained from the same embeddings and folds. Fusion produced a modest but consistent improvement over the best single modality, and simpler fusion schemes outperformed more expressive ones.

As shown in Table 5, the best fusion strategy exceeded the best single modality on every grading task, but the gains were small. On the five-class task, late fusion reached a macro-F1 of 0.470, compared with 0.441 for WSI-only and 0.373 for RNA-only within the same pipeline, an improvement of 0.029 over the stronger modality. The gain was similar on the three-class formulations (frozen late fusion: 0.684 versus 0.648 WSI-only for the 1/23/45 grouping; gated fusion: 0.668 versus 0.625 for the 12/3/45 grouping) and on the binary task (gated fusion: 0.826 versus 0.802). Critically, the more expressive strategies did not help: low-rank tensor fusion and cross-attention consistently underperformed the simpler late and gated schemes, with tensor fusion falling to 0.410 on the five-class task, below even the WSI-only baseline. This pattern is consistent with overfitting of high-capacity interaction models with a cohort of a few hundred patients.

The staging endpoints revealed that the value of fusion is itself task-dependent, mirroring the unimodal contrast. Under repeated stratified five-fold cross-validation, the best combination for N-stage was Gleason grade plus RNA, reaching an AUROC of 0.772, a significant improvement over the strongest single modality (Δ = +0.077 over grade alone; bootstrap 95% CI: +0.031 to +0.128; permutation *p* < 0.001), whereas adding the WSI modality to the molecular signal was not beneficial (WSI-plus-RNA reached 0.673, not significantly above the best single modality; Δ = −0.019, *p* = 0.69). For T-stage, the significant gains instead came from adding morphology, with the grade-plus-WSI combination performing best (AUROC: 0.805; Δ = +0.037 over WSI alone, *p* = 0.026), while RNA added little. As noted in Section 3.3, these staging increments are computed against the fusion analysis’s own re-estimated unimodal baselines, which differ slightly from the primary unimodal estimates in Table 4. Thus, morphology contributes to fusion for local tumor extent but degrades it for nodal status, so that the optimal modality combination is not fixed but depends on the endpoint.

These fusion comparisons constitute an explicit modality-ablation analysis: they measure the marginal effect of adding one modality to the other. Adding transcriptomics to grade improved nodal prediction (+0.077 AUROC), whereas adding morphology to the molecular signal did not help and slightly degraded it for nodal status, while the reverse held for T-stage. Because the marginal contribution of a modality is thus positive for one endpoint and null or negative for another, the multimodal benefit cannot be attributed to a single globally dominant modality. We emphasize that the central claim of this study rests on the unimodal contrast rather than on the fusion gain: each modality was evaluated on its own (WSI-only and RNA-only), and it is the reversal of their relative standing across endpoints, established statistically in Section 3.1, that supports task-dependence. Consistent with this, the fusion gains are modest throughout, so the concern that an apparent multimodal improvement might arise without genuine patient-level correspondence does not affect our conclusions; the significance of the observed contrast was in any case confirmed by label-permutation tests (nodal gap *p* = 0.037; reversal interaction *p* = 0.052) rather than inferred from fusion performance.

We repeated the full fusion comparison with the standard (non-gated) ABMIL encoder and observed the same qualitative pattern, so the conclusions do not depend on the choice of attention encoder. With ABMIL, the best fusion strategy again provided only a modest gain over the stronger unimodal baseline (for example, a five-class macro-F1 of 0.449 for late averaging versus 0.429 for WSI-only), the more expressive cross-attention scheme again underperformed simpler late and concatenation fusion on every task (for example, 0.432 versus 0.449 on the five-class task), and RNA-only fusion remained the weakest configuration throughout. The best fusion strategy varied slightly between the two encoders across tasks (late averaging, concatenation, or frozen late fusion), but in all cases simple fusion schemes matched or exceeded the most expressive ones, confirming that the modest, task-dependent character of the fusion gain is a property of the data rather than of a single encoder. Formal significance testing of fusion gains was applied to the staging endpoints, where the modality contrast is the primary claim; for the grading tasks, differences between fusion strategies were small relative to cross-validation variability and are reported descriptively.

### 3.3. Robustness and Biological Basis of the Contrast

The morphology–molecule contrast could in principle be an artifact of how transcriptomics was modeled, of tumor purity, of batch effects, or of a simple correlation with grade. We therefore subjected the molecular findings to a series of controls before interpreting them biologically. None of these controls overturned the contrast, and a gene set enrichment analysis connected the transcriptomic nodal signal to established prostate stromal biology.

First, to ensure that morphology’s advantage in grading was not an artifact of underpowered RNA modeling, we compared all seven RNA configurations. On the five-class task, RNA macro-F1 ranged only from 0.30 to 0.40 across principal component analysis, supervised and unsupervised gene selection, regularized linear models, and gradient boosting, with the strongest configuration (0.398) still below the fixed morphological model (0.445). Gradient boosting was the weakest, consistent with the difficulty of tree ensembles on high-dimensional, small-sample expression data. The same ordering held on the finer-grained grading tasks, where morphology led on every ordinal metric; the sole exception was the binary high-grade task, where the best RNA configuration matched morphology, as noted in Section 3.1. This confirms that the morphological advantage on the graded tasks is robust to the RNA modeling choice rather than an artifact of a single pipeline (Table 6).

The low absolute accuracy of RNA on the five-class grading task (for example, 0.466) is therefore not a limitation of our modeling but an expected and central component of the task-dependent finding: transcriptomics carries only weak information about a morphologically defined quantity such as Gleason grade, even as the same RNA representations achieve strong performance for nodal status (Section 3.1). Grade is read from glandular architecture, which is directly accessible to imaging but only indirectly reflected in bulk expression, so the modest RNA grading accuracy is consistent with the biology rather than indicative of an underpowered pipeline. Conversely, the morphological predictability of grade is expected on established histopathological grounds: Gleason grade is defined by glandular architecture, precisely the tissue feature that image-based models access directly, so a strong morphological signal for grade reflects the accepted determinant of prostate cancer aggressiveness rather than a spurious correlation.

Table 6 lists the seven RNA configurations. The spread across methods was small relative to the gap to morphology, and the best RNA result remained below the corresponding morphological result on the graded (non-binary) tasks reported in Table 3. Notably, this stability also indicates that RNA performance is bounded by the information available in the data rather than by the choice of model, a point we return to in Section 4.

Second, we addressed three confounders specific to the transcriptomic N-stage signal. Tumor purity was not responsible: the correlation between an ESTIMATE-based purity score and N-stage was negligible (point-biserial r = −0.03), and the RNA signal persisted after purity adjustment. Batch effects were not responsible: the association between tissue source site and N-stage was not significant according to a chi-square test, indicating independence from the multi-institutional structure of the cohort. Finally, the molecular signal was not merely a proxy for grade: adding the transcriptomic score on top of Gleason grade produced a significant increment in nodal AUROC (Δ = +0.077, bootstrap 95% CI: +0.031 to +0.128, permutation *p* < 0.001), showing that RNA carries nodal information beyond what grade encodes. The corresponding increment for T-stage was smaller (Δ = +0.029, 95% CI: +0.006 to +0.052, *p* = 0.004), consistent with morphology already capturing most of the local-extent signal. These increments come from a stacked fusion analysis whose internally re-estimated unimodal baselines differ slightly from the primary unimodal AUROCs in Table 4, as expected for two independent cross-validation runs. The symmetric question for morphology, whether it retains predictive value for grade after adjusting for clinical variables, is largely definitional, since Gleason grade is itself the pathological variable in question and is defined by the glandular architecture that morphology reads. We did not attempt to adjust the nodal analysis for additional clinical covariates beyond grade, because with only 47 node-positive cases such multivariable adjustment would be underpowered; we therefore report the grade-adjusted result as the primary confounder control and treat further adjustment as beyond the statistical resolution of this cohort.

**Figure 4:**
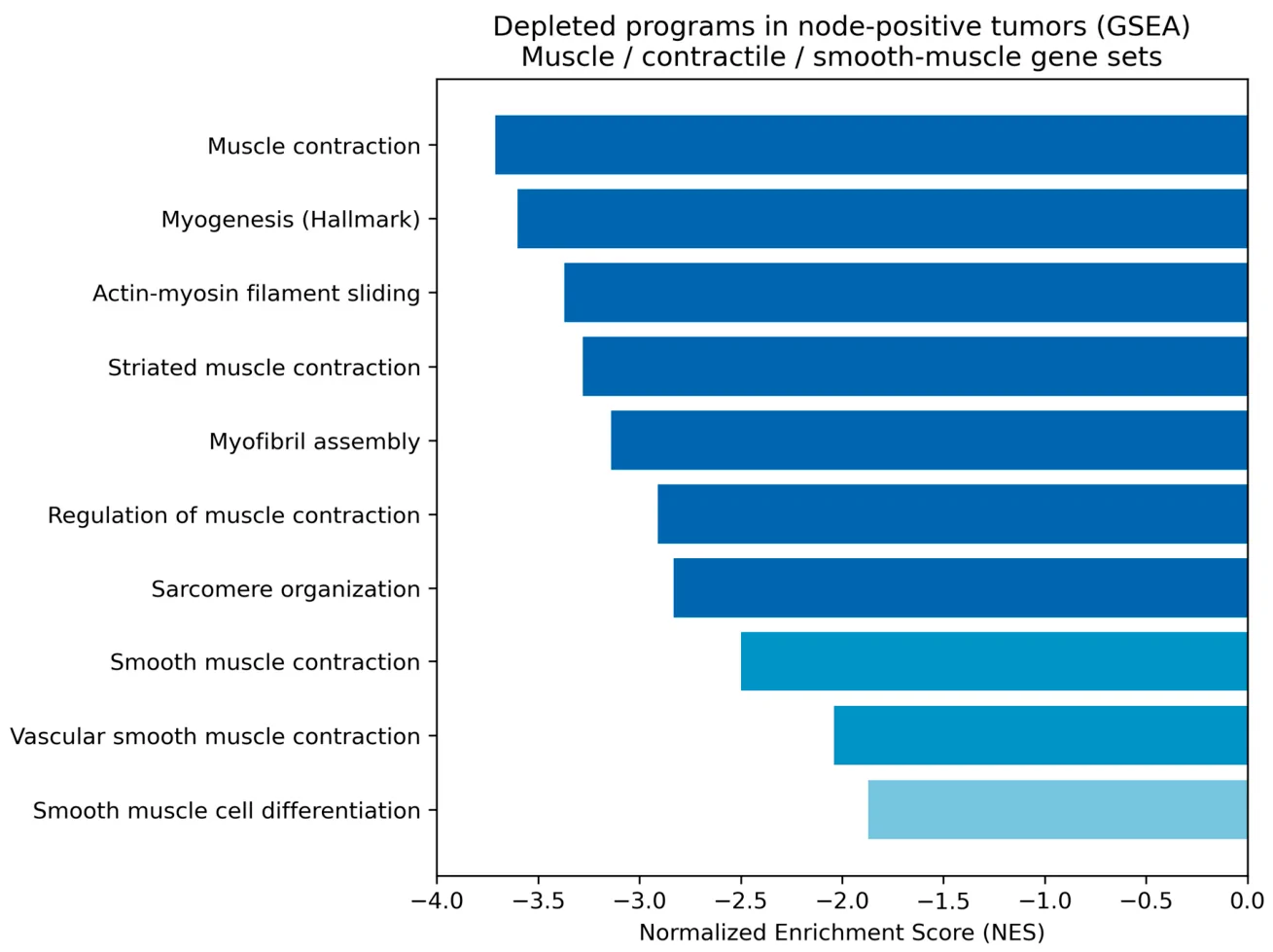
Loss of muscle and contractile programs in node-positive tumors. Gene set enrichment analysis (GSEA) of the differential expression between node-positive and node-negative tumors, showing the ten most strongly depleted muscle, contractile, and smooth muscle gene sets ranked by normalized enrichment score (NES). All displayed gene sets are significantly depleted in node-positive disease (all false discovery rate q < 0.001), led by muscle contraction (NES = −3.71) and myogenesis (NES = −3.60), and including smooth muscle contraction and differentiation. This coordinated loss of smooth muscle and contractile programs is the transcriptomic signature of the reactive-stroma transition and dedifferentiation that accompany prostate cancer progression, a well-established biology that provides a mechanistic basis for why nodal status is legible to transcriptomics but not to morphology.

Having excluded these artifacts, we asked what molecular program underlies the nodal signal. Gene set enrichment analysis of the differential expression between node-positive and node-negative tumors showed a strong, coordinated loss of smooth muscle and contractile programs in node-positive disease, with muscle-contraction gene sets among the most negatively enriched (the normalized enrichment score was approximately −3.7, and the false discovery rate was near zero) (Figure 4). This pattern matches the well-documented reactive-stroma transition and smooth muscle dedifferentiation that accompany prostate cancer progression. We emphasize that this biology is established rather than newly discovered here; our contribution is to show that the same molecular program explains why nodal status is legible to transcriptomics but not to morphology. The reproducible nodal signature (genes retained in at least 80% of inner folds) was dominated by stromal remodeling, extracellular matrix, matrix metalloproteinase, and chemokine genes, consistent with the reactive-stroma and dedifferentiation program identified by the enrichment analysis. Notably, the signature contained no Y-chromosome genes, confirming that the male-only cohort did not introduce a sex-linked technical artifact into the nodal signal.

Figure 4 shows the enrichment profile for the leading muscle-contraction and myogenesis gene sets. The consistent negative enrichment in node-positive tumors provides a coherent mechanistic account of the modality contrast: metastatic propensity is encoded in a stromal gene-expression state that is only weakly reflected in glandular architecture and is therefore accessible to transcriptomics but largely invisible to image-based models. We interpret this program as a predictive association rather than a demonstrated causal mechanism: the transcriptomic state is informative about nodal status, but our observational, cross-sectional data cannot establish that the loss of contractile programs causes metastatic spread.

We also examined how performance and modality contribution vary across grade groups and nodal categories, and the characteristic failure modes of each modality. Performance by grade group is captured directly by the multi-formulation results (Figure 2, Table 3): morphological accuracy is highest for the binary high-grade distinction and lowest for the five-class formulation, where errors are concentrated between adjacent grade groups, consistent with the low mean absolute error on the ordinal scale despite a nonzero rate of severe (two-grade) errors. For nodal status, the two modalities differ mainly in specificity and precision rather than sensitivity: at the Youden-optimal threshold, both reach a sensitivity of 0.62, but the transcriptomic model attains a higher specificity (0.73 versus 0.59) and precision (0.28 versus 0.21), which is the origin of its higher PR-AUC and balanced accuracy (Table 4). The direction of the task-dependent contrast was consistent when the cohort was split coarsely by grade, but we deliberately did not pursue finer subgroup analyses of nodal prediction: with only 47 node-positive patients, any further stratification yields subgroups too small for reliable estimation, and we report this limit rather than over-interpret underpowered subgroups.

### 3.4. Uncertainty Quantification and Protocol Comparison

Two practical questions remain for deployment. First, can the grading model express calibrated uncertainty, so that ambiguous cases are flagged rather than forced into an overconfident label? Second, how much does the choice of evaluation protocol, a single locked test versus repeated cross-validation, affect the apparent performance and modality contributions in a cohort of this size? We addressed the first with conformal prediction on the locked protocol and the second by comparing the two protocols directly.

Conformal prediction with adaptive prediction sets, calibrated on the held-out calibration partition of the locked protocol, achieved empirical coverage close to the target level while producing informative prediction sets (Table 7). On the three-class grading task at a target coverage of 0.90, the empirical coverage was 0.902 with a mean prediction-set size of 1.76 out of three classes, so that confident cases were resolved to a single grade group while ambiguous cases retained two. The five-class task, being harder, required larger sets (mean: 3.07 at 0.90 coverage), consistent with the greater difficulty of resolving five ordinal grades. In both cases, the coverage guarantee held, providing a per-case, interpretable measure of reliability that is directly usable in clinical triage, where large prediction sets can be routed for expert review.

Finally, the two evaluation protocols diverged substantially, which is itself a finding about small-cohort evaluation. On the locked single test partition of 41 patients, RNA-only reached a five-class QWK of 0.838, while WSI-only reached only 0.444, which taken alone would suggest that transcriptomics dominates grading. Cross-validation reversed this picture: across all patients, WSI-only reached a QWK of 0.613 and the best RNA configuration of 0.591, restoring the expected morphological advantage. The locked estimates are not wrong, but a single test partition of about forty patients is small enough that an unrepresentative draw can distort ordinal metrics that depend on a handful of cases, in this instance inflating RNA and depressing WSI. Because the locked and cross-validation protocols also differed in configuration, we do not treat their difference as a controlled comparison; nonetheless, the reversal illustrates concretely that a single locked test can invert the apparent ranking of modalities in a cohort of this size. Reporting both protocols therefore gives a more honest picture: the locked protocol anchors an unbiased final number and the conformal guarantee, while cross-validation provides the stable modality-contribution estimates on which our main conclusions rest.

## 4. Discussion

The central finding of this study is that morphology and transcriptomics contribute unequally, and in a task-dependent way, to the two clinically central endpoints of prostate cancer. Gleason grade was best predicted from morphology: a single fixed attention-based model on UNI features exceeded the best of seven RNA configurations on the finer-grained grading tasks, and this advantage held across all 45 morphological configurations rather than being confined to a favorable model. Nodal status showed the opposite pattern: no morphological configuration exceeded an AUROC of 0.62, with most near chance, whereas transcriptomics reached 0.68 and carried nodal information beyond what grade alone encodes. T-stage sat between these extremes, predicted comparably by both modalities. This is not a claim that one modality is generally superior, but that the informative modality depends on the biological nature of the endpoint, a distinction that is easily obscured when studies report only aggregate fusion performance.

The task-dependent contrast has a coherent biological interpretation, and it aligns with prior evidence rather than contradicting it. Gleason grade is by construction a description of glandular architecture, so its predictability from images is expected. Nodal metastatic propensity, by contrast, reflects a stromal and microenvironmental state. Our gene set enrichment analysis showed a strong, coordinated loss of smooth muscle and contractile programs in node-positive tumors, consistent with the well-documented reactive-stroma transition and smooth muscle dedifferentiation that accompany prostate cancer progression. We stress that this biology is established rather than newly discovered here; several transcriptomic and single-cell studies have linked stromal remodeling and differentiation-related programs [28,29,30] to metastasis and adverse outcomes in prostate cancer. Our contribution is to connect this molecular program to a concrete predictive asymmetry, explaining why nodal status is legible to transcriptomics but not to image-based grading.

This account is also consistent with prior imaging studies. Wessels et al. [27], using a convolutional neural network on a Gleason-matched cohort, reached only a mean AUROC of 0.68 with 53% sensitivity for nodal metastasis and missed roughly half of node-positive cases. Rather than contradicting our result, that study reinforces it: even a dedicated imaging model, after controlling for grade, recovers only a weak morphological signal for nodal status. Our finding that 45 diverse morphological configurations all remain near chance for N-stage extends this observation from a single architecture to a broad model space.

For Gleason grading, our morphological performance is consistent with prior work on the same TCGA-PRAD cohort. Otálora et al. [35] compared weakly supervised strategies for five-class Gleason grading on TCGA-PRAD whole-slide images using only slide-level report labels, the same weak-supervision setting used here, and reported that finer-grained five-class grading remains substantially harder than binary low-versus-high discrimination. Our results reproduce this pattern: morphological performance is strong for the binary high-grade task and degrades on the five-class formulation, where the class-balanced macro-F1 is necessarily lower. The purpose of our study is not to surpass a dedicated grading model but to place morphology and transcriptomics on a common, leakage-controlled footing across the same endpoints, so that their relative contributions can be compared directly rather than in isolation.

Positioned against the three established lines of work, our study occupies a distinct niche. Image-based grading models have reached expert-level Gleason classification [16,17,35], and transcriptomic signatures have been developed for prognosis and recurrence [19], while multimodal fusion frameworks have combined histology and molecular data primarily to maximize a single prognostic or survival endpoint [21,22,23,24]. Our aim is different: rather than proposing a new architecture or a new signature, we hold the modeling deliberately simple and standardized and use it as an instrument to ask which modality is informative for which endpoint. The novelty is therefore the systematic, leakage-controlled demonstration that modality contribution is task-dependent, together with a biological rationale for the contrast and a direct statistical test of the reversal, rather than a claim of state-of-the-art performance on any single task. To our knowledge, this task-dependent asymmetry between morphology and transcriptomics across grade and nodal status has not previously been isolated and quantified on a common cohort.

Two methodological observations follow. First, multimodal fusion provided only modest gains over the stronger single modality on the grading tasks, and more expressive fusion architectures, including cross-attention and low-rank tensor interaction, underperformed simpler late and gated schemes, in several cases falling below the unimodal baseline. At a cohort of a few hundred patients, high-capacity interaction models appear to overfit, and the value of fusion is itself task-dependent: adding morphology improved nodal prediction not at all and in fact degraded it, whereas it helped for local tumor extent. Fusion should therefore be reported against matched unimodal baselines and interpreted per endpoint, rather than as a single headline number.

Two conceptual distinctions also follow. First, predictive usefulness and modality importance are not the same: a modality may carry complementary information for one endpoint while adding noise for another, so modality importance must be assessed per task rather than globally. Second, because the optimal modality combination differs by endpoint, morphology helping for T-stage but not for nodal status, these results argue against a single shared multimodal architecture and in favor of systems that select or weight modalities according to the clinical prediction task.

The second methodological observation concerns the evaluation protocol: the two protocols diverged in a way that is instructive for small-cohort studies. On the single locked test partition, RNA appeared to dominate grading, with a five-class QWK of 0.838 versus 0.444 for morphology; cross-validation reversed this, restoring the morphological advantage (0.613 versus 0.591). A single test set of about forty patients is simply too small to rank modalities reliably on ordinal metrics. Although the locked and cross-validation pipelines differed in configuration and so do not constitute a controlled comparison, the reversal is a concrete demonstration that small locked test sets can invert apparent modality rankings. We therefore regard the dual-protocol design, reporting an unbiased locked estimate alongside stable cross-validated contribution estimates, as a methodological safeguard rather than a redundancy.

Clinically, the results suggest a pragmatic division of inputs. Where the question is grade, high-quality histology with a strong foundation model and a simple attention aggregator is sufficient and does not require transcriptomics. Where the question is nodal or metastatic risk, morphology alone is inadequate and molecular profiling adds genuine information. The conformal prediction layer makes this actionable: at 90% target coverage, the three-class grading model produced prediction sets averaging 1.76 of three grade groups, so that confident cases receive a single label while ambiguous cases are flagged with larger sets that can be routed for expert review. Such calibrated abstention is particularly valuable for the minority high-grade and node-positive cases, where errors are most consequential.

Several limitations qualify these findings. First, the study used a single cohort (TCGA-PRAD) without external validation, so the absolute performance figures and the exact contribution estimates may not transfer to other populations or scanners; the task-dependent direction of the contrast, however, rests on a broad configuration grid and is unlikely to be an artifact of a single model. External validation is the most important next step, and it would need to address several sources of domain shift: differences in histology staining and slide preparation, whole-slide scanner characteristics, and RNA sequencing platforms and transcriptomic preprocessing across institutions, all of which can affect both the foundation-model features and the transcriptomic representations. We frame the absolute performance figures as cohort-specific and the task-dependent contrast as a hypothesis-generating finding that requires confirmation on independent, multi-institutional cohorts with matched imaging and transcriptomics. No suitable external cohort with matched whole-slide images and RNA sequencing for these endpoints was available to us, so external validation remains a goal for future work. The study is also retrospective and single-source, and the N-stage analysis is restricted to the 316 patients who underwent lymph node dissection, which may introduce selection bias; the observed modality specialization may therefore be partly dataset-dependent. Second, nodal status could be evaluated in only 316 patients, of whom 47 were node-positive, which limits statistical power; this is why the endpoint-by-modality interaction, although robust in direction (bootstrap 95% CI excluding zero), is only of moderate statistical strength (permutation *p* = 0.052). This limited number of node-positive cases also precludes reliable subgroup analysis of nodal prediction, for example, by grade group or tumor burden, so we restrict nodal claims to the whole evaluable cohort. Third, the locked and cross-validation protocols differed in configuration, so their comparison illustrates rather than quantifies the effect of protocol choice. Fourth, fusion was restricted to UNI features and two attention encoders to bound computation, so we cannot exclude that other feature-encoder combinations would fuse differently, though the unimodal analysis makes a large change unlikely. Our interpretation of the morphological signal rests on the definitional link between Gleason grade and glandular architecture rather than on an explainability analysis; we did not perform attention-map or saliency visualization to confirm that the models attend to the expected tissue features, which remains a useful direction for future work. Finally, bulk RNA sequencing averages over the tumor and its microenvironment; spatial or single-cell profiling might localize the stromal signal we detect and further sharpen the biological interpretation.

## 5. Conclusions

We presented a rigorously evaluated multimodal analysis of prostate cancer on the TCGA-PRAD cohort, designed to characterize how morphology and transcriptomics contribute to different clinical endpoints rather than to maximize a single performance metric. The central result is a task-dependent division of labor: Gleason grade, a morphologically defined endpoint, was best predicted from whole-slide images, with a single fixed attention-based model exceeding the best of seven transcriptomic configurations and the advantage holding across all 45 morphological configurations; nodal status, by contrast, was predicted by transcriptomics but not reliably by morphology, with no configuration exceeding an AUROC of 0.62 and most remaining near chance. T-stage was intermediate, captured comparably by both modalities. A gene set enrichment analysis connected the transcriptomic nodal signal to the established loss of smooth muscle and contractile programs in prostate cancer progression, providing a biological rationale for the contrast rather than a novel mechanism. Two methodological findings support these conclusions. Multimodal fusion yielded only modest, task-dependent gains, with simpler schemes outperforming more expressive interaction models at this cohort size and with morphology improving fusion for local tumor extent but degrading it for nodal status. A dual evaluation protocol proved essential: a single locked test set inverted the apparent modality ranking for grading, which repeated cross-validation corrected. Finally, conformal prediction added calibrated, per-case uncertainty suitable for clinical triage. Taken together, these results argue that the informative modality in prostate cancer classification is not fixed but depends on the biological nature of the endpoint and that honest characterization of modality contributions requires both broad configuration coverage and stable evaluation. Validating this task-dependent structure on external cohorts and localizing the stromal signal with spatial or single-cell profiling are natural next steps.

## Figures and Tables

**Table 1:** Cohort characteristics and classification endpoints (TCGA-PRAD, *n* = 401). All 401 patients have matched whole-slide images and RNA-seq; T-stage and N-stage were evaluable in the subsets with available pathologic staging (*n* = 373 and *n* = 316, respectively).

Endpoint	Formulation	Class Distribution (*n*)	Evaluable *n*
Gleason Grade Group	5-class	40/121/89/50/101	401
Gleason Grade Group	3-class (1/23/45)	40/210/151	401
Gleason Grade Group	3-class (12/3/45)	161/89/151	401
Gleason Grade Group	binary (high-grade)	250/151	401
T-stage	binary	149/224	373
N-stage	binary	269/47	316

Abbreviations: GG, grade group. T3+ includes pathologic T3 and T4. N-stage labels were available only for patients who underwent lymph node dissection.

**Table 2:** Feature extraction and configuration grid for each modality.

Modality	Component	Options
Morphology (WSI)	Foundation model	UNI (1024-d); Virchow2 (2560-d); Phikon (768-d); Phikon-v2 (1024-d); H-optimus-0 (1536-d)
	Aggregation	Pooling: mean, max, top-k mean, std; Attention MIL: ABMIL, gated ABMIL, CLAM; Dual-stream: DSMIL; Transformer: TransMIL
	Configurations per task	per task 5 foundation models × 9 aggregators = 45
Molecular (RNA)	Preprocessing	log2(TPM + 1), training-fold low-expression filtering
	Configurations (representation & classifier)	PCA-128, MLP; ANOVA top-2000 + MLP; high-variance top-2000 + MLP; full genes + L2 logistic regression; PCA-128 + XGBoost; ANOVA top-2000 + XGBoost; full genes + elastic-net
	Total	7 representation–classifier combinations
Fusion	Inputs	WSI slide embedding (ABMIL or gated ABMIL, UNI features) + matched RNA embedding (PCA-128 + MLP)
	Fusion strategy	concatenation; late (probability averaging); gated; cross-attention; frozen late; low-rank tensor (Kronecker)
	Total	2 WSI encoders × 5 strategies + 1 tensor (best encoder) = 6 strategies, with WSI-only and RNA-only baselines

Abbreviations: WSI, whole-slide image; MIL, multiple instance learning; PCA, principal component analysis; ANOVA, analysis of variance; TPM, transcripts per million.

**Table 3:** Unimodal performance on the Gleason grading tasks (repeated stratified five-fold cross-validation). A single fixed morphological configuration (gated ABMIL on UNI features) is compared against the best RNA configuration per task.

Task	Modality	Accuracy	Macro-F1	QWK	MAE	Severe	MCC
5-class	WSI	0.501	0.445	0.613	0.756	78	0.285
	RNA	0.466	0.398	0.591	0.805	75	0.297
3-class (1/23/45)	WSI	0.726	0.637	0.619	0.277	1	0.521
	RNA	0.721	0.618	0.536	0.297	7	0.497
3-class (12/3/45)	WSI	0.648	0.603	0.633	0.426	30	0.455
	RNA	0.651	0.599	0.606	0.444	38	0.455
Binary (high-grade)	WSI	0.815	0.799	n/a	0.185	0	0.622
	RNA	0.815	0.802	n/a	0.185	0	0.604

A single WSI configuration is shown for all tasks to avoid selecting the best morphological model per task; RNA is the strongest of the seven configurations per task. Morphology leads on the ordinal metrics (macro-F1 and QWK) for the finer-grained tasks; on the five-class task, the two modalities are essentially tied under MCC (0.285 versus 0.297), and on the binary task RNA is marginally higher on macro-F1. QWK undefined for binary.

**Table 4:** Unimodal AUROC for staging endpoints (repeated stratified five-fold cross-validation). Threshold-dependent metrics (sensitivity, specificity, precision, F1, and balanced accuracy) use the Youden-optimal threshold. The random-baseline PR-AUC equals the positive-class prevalence (0.149 for N-stage, 0.601 for T-stage). For the WSI modality, staging used a PCA-reduced logistic model on UNI features, a single representative configuration distinct from the 45-configuration grid in Figure 3; Gleason grade denotes the pathologic grade group used as a predictor.

**N-Stage (*n* = 316)**							
**Modality**	**AUROC**	**PR-AUC**	**Sens**	**Spec**	**Prec**	**F1**	**bAcc**
Gleason grade	0.687	0.226	0.79	0.68	0.30	0.44	0.74
WSI (UNI)	0.574	0.199	0.62	0.59	0.21	0.31	0.60
RNA (full PCA)	0.683	0.260	0.62	0.73	0.28	0.39	0.67
**T-Stage (*n* = 373)**							
**Modality**	**AUROC**	**PR-AUC**	**Sens**	**Spec**	**Prec**	**F1**	**bAcc**
Gleason grade	0.741	0.781	0.74	0.73	0.80	0.77	0.73
WSI (UNI)	0.770	0.844	0.74	0.67	0.77	0.76	0.71
RNA (full PCA)	0.716	0.789	0.78	0.54	0.72	0.75	0.66

For N-stage, the WSI confidence interval includes 0.5 (chance), whereas RNA does not. For T-stage, all three modalities exceed chance.

**Table 5:** Fusion strategies versus unimodal baselines on the Gleason grading tasks (macro-F1, repeated stratified five-fold cross-validation; gated ABMIL on UNI features fused with a matched RNA embedding).

Fusion Strategy	5-Class	3-Class (1/23/45)	3-Class (12/3/45)	Binary
Concatenation	0.453	0.678	0.632	0.823
Late	0.470	0.668	0.625	0.811
Gated	0.405	0.673	0.668	0.826
Cross-attention	0.442	0.648	0.609	0.795
Frozen late	0.432	0.684	0.646	0.820
Low-rank tensor	0.410	0.597	0.609	0.817
WSI-only (baseline)	0.441	0.648	0.625	0.802
RNA-only (baseline)	0.373	0.581	0.558	0.783

Unimodal baselines are computed from the same embeddings and folds. Values are macro-F1; QWK follows a similar overall pattern, with fusion exceeding the unimodal baselines and the more expressive strategies underperforming.

**Table 6:** RNA-only performance across the seven configurations on the five-class Gleason task (macro-F1, repeated stratified five-fold cross-validation).

Feature Representation	Classifier	Macro-F1
ANOVA top 2000	MLP	0.398
Full genes	L2 logistic	0.385
Full genes	elastic-net	0.375
PCA-128	MLP	0.367
ANOVA top 2000	XGBoost	0.356
High-variance top 2000	MLP	0.344
PCA-128	XGBoost	0.304

All seven RNA configurations fall between 0.30 and 0.40 on the five-class task, below the fixed morphological model (macro-F1: 0.445; Table 3). Gradient boosting (XGBoost) is weakest.

**Table 7:** Conformal prediction on the locked test set (*n* = 41) using adaptive prediction sets. Empirical coverage and mean prediction-set size at two target coverage levels.

Task	Target Coverage (1 − α)	Empirical Coverage	Mean Set Size
5-class	0.90	0.951	3.07
5-class	0.80	0.878	2.20
3-class	0.90	0.902	1.76
3-class	0.80	0.854	1.59

Adaptive prediction sets calibrated on the locked calibration partition (*n* = 40). Empirical coverage is close to the target at each level; mean set size indicates how many grade groups are retained on average. Smaller sets at fixed coverage indicate a more confident predictor. On the three-class task, the mean set size of 1.76 (out of three classes) shows that most cases are resolved to one or two grade groups.

## Data Availability

The original data presented in the study are openly available. The clinical annotations and bulk RNA-sequencing data are available in the Genomic Data Commons (GDC) Data Portal at https://portal.gdc.cancer.gov (accessed on 12 September 2026) under the project accession TCGA-PRAD. The diagnostic whole-slide images are available in The Cancer Imaging Archive (TCIA) at https://doi.org/10.7937/K9/TCIA.2016.YXOGLM4Y (accessed on 12 September 2026).
